# Nanopesticides for managing primary and secondary stored product pests: Current status and future directions

**DOI:** 10.1016/j.heliyon.2025.e42341

**Published:** 2025-01-30

**Authors:** Mohammed Lengichow Kadir, Asli Dageri, Tuğba Nur Aslan

**Affiliations:** a Department of Biology, College of Natural and Computational Science, Wolkite University, Wolkite, Ethiopia; b Department of Molecular Biology and Genetics, Necmettin Erbakan University, Meram, Konya, 42090, Turkey

**Keywords:** Biosafety, Nanoparticles, Nanopesticides, Oxidative stress, Pest management, Post-harvest losses, Storage pest

## Abstract

The preservation of agricultural commodities during storage is critical for ensuring food security and minimizing post-harvest losses. Both primary storage pests such as *Callosobruchus maculatus*, *Callosobruchus chinensis*, *Sitophilus weevils*, *Rhyzopertha dominica*, and *Trogoderma granarium*, and secondary storage pests like *Tribolium castaneum* cause significant damage to stored products, resulting in substantial economic losses. Traditional pest control methods, including chemical insecticides, face limitations due to environmental concerns and pest resistance. Consequently, nanoparticle-based insecticides are being extensively suggested as a promising alternative. This review analyzes the available literature on the efficacy of nanoparticles (NPs) against primary and some secondary storage pests. Green synthesis methods using plant extracts and other biological sources are highlighted for the production of environmentally friendly NPs. Studies demonstrate that NPs of alumina, carbon, silica, silver, copper, zinc oxide, nickel oxide, titanium dioxide, nano zeolite, as well as chitosan and polymers exhibit significant insecticidal activity against a variety of pests, in some cases surpassing mortality rates caused by traditional insecticides at recommended dosages. Structural, biochemical and molecular studies reveal that NPs induce oxidative stress, disrupt cellular homeostasis, and cause structural damage in pests. Histopathological evaluations indicate specific organ-related toxicity, emphasizing the need for comprehensive biosafety assessments. Additionally, the integration of NPs with conventional insecticides shows enhanced pest control efficiency, although challenges remain in standardizing synthesis methods and evaluating long-term environmental impacts. This review highlights the potential of NPs in sustainable pest management and underlines the importance of ongoing research to optimize specific formulations for specific groups of pests and ensure safety.

## Highlights

• Green NPs are being increasingly studied for insecticidal use against storage pests.
• NPs affect cellular functions, cause oxidative stress and structural damage in pests.
• NPs applied to plants are safe and can enhance certain agronomic traits.
• Studies suggest variability in NPs synthesis methods.
• Varied application methods may affect reproducibility in pest control efficacy.

## Graphical abstract

**Figure:**
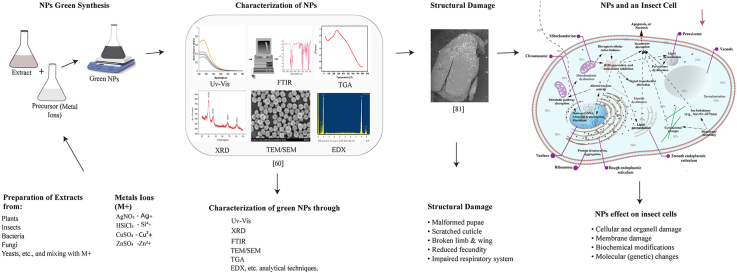

## 1 Introduction

The storage of agricultural products for postharvest utilization from one season to the next is one of the most ancient human practices, dating back thousands of years to the Pre-Pottery Neolithic era [1]. Stored products, whether of agricultural or animal origin, and whether edible or non-edible, durable or fresh, raw or processed, are susceptible to pest attacks at various stages from production to consumption [2]. Pests that affect agricultural production include insects primarily from the orders Coleoptera and Lepidoptera, as well as arachnids such as ticks and mites. In particular, insect pests of stored products pose a significant challenge by reducing both the quantity and quality of crop yields and potentially threatening plant, animal, and human health [3]. These pests can lead to yield losses in cereals, legumes, vegetables, seeds, nuts, and industrial crops such as cotton and millet. The khapra beetle, for instance, is reported to infest over 100 types of stored products, including dried fruits such as figs, raisins, peaches, prunes, and dates; processed items like cassava chips and egg noodles; various animal or plant products; and even dead insects [4]. Further exacerbating the challenge is the ability of storage pests, such as the khapra beetle, to enter prolonged diapause lasting several years. This adaptive strategy enables them to evade control measures, endure adverse conditions, and significantly complicate pest management efforts [5].

Postharvest losses from certain stored-product insect pests and phatogens reach as high as 20 %–40 % each year [6], and sometimes to 100 % [7,8]. The loss can be classified into direct (active) and indirect (passive) impacts on agriculture. Directly, pest infestations cause economic losses by restricting trade, posing health risks, and rendering produce unfit for human consumption [7]. During seasonal storage, contamination of wheat grains with 15 % khapra beetle caused loss of weight and viability of around 2.6 % and 24 %, respectively [9]. Furthermore, pest infestation of grains leads to significant damage to nutrient contents, such as gluten, vitamins, fats, and sugars, by consuming the dry matter of crops [10]. For instance, a study conducted on the impact of wheat grain infestation by the boring pest *Sitophilus zeamais* (*S. zeamais*) during postharvest storage revealed a decrease in protein quality by altering its composition and structural properties. Additionally, the gluten network structure of the protein was disrupted, the color and textural properties of whole wheat noodles were affected [11]. Indirectly, pest infestation in stored grains can alter moisture content and temperature, which can lead to the growth of antibiotic-resistant pathogens, potentially turning the grains into reservoirs of disease. Left over webs, exuviae, frass, and cadavers from the infesting pests affect the physical qualities of the stored products [12,13]. Given the significant economic losses and health risks posed by these pests, it is crucial to implement various effective pest management strategies to protect both the quality and quantity of stored agricultural products.

To mitigate and alleviate pest infestations in agriculture, various methods have been employed, including chemical and microbial insecticides; temperature and radiation treatments; botanicals; natural enemies; pheromones, including semiochemicals; insect growth regulators; molecular approaches; transgenic crops; and, more recently, nanoparticles (NPs) [14,15]. Commonly used conventional control methods, such as insecticides and fumigants, are not only environmentally unfriendly but also adversely affect seed germination and contaminate grains with toxic pesticide residues [16]. In contrast, NPs, also referred to as nanopesticides in insect control, have emerged as a promising alternative for managing stored product pests, offering several advantages over conventional insecticides. These include enhanced chemical solubility, lower dosage requirements, controlled release of active ingredients, and reduced environmental toxicity [17,18]. NPs synthesized using biological agents also provide benefits such as ease of handling, resource availability, and higher productivity, making them a safer and more sustainable solution for pest control. Additionally, their integration into pest management strategies could revolutionize agriculture by improving crop yields, reducing reliance on harmful chemical pesticides, and mitigating the negative side effects of phased-out pesticides [19,20]. However, a comprehensive review of the literature on the efficacy of these NPs against primary and secondary storage pests is lacking. This article aims to consolidate existing literature on the effectiveness of NPs specifically against primary storage pests and some secondary storage pests.

## 2 Control approaches against stored product insects

Stored product insects can be categorized as primary and secondary pests based on their feeding habits and the extent of damage they cause. Primary storage pests, such as the lesser grain borer *R. dominica* F. (Coleoptera: Bostrychidae), the grain weevil *S. granarius* L. (Coleoptera: Curculionidae), the pulse beetle *C. chinensis* (Coleoptera: Bruchidae), and the khapra beetle *T. granarium* Everts (Coleoptera: Dermestidae) consume whole, undamaged grains and can cause extensive losses to stored grain lots if left unaddressed early [21]. On the other hand, secondary storage pests such as red flour beetle *T. castaneum* Herbst (Coleoptera: Tenebrionidae), potato tuber moth *Phthorimaea operculella* and the rice moth *Corcyra cephalonica* attack the grains that have already been damaged by primary pests or other factors. Hence, pests under this category thrive on broken kernels, grain dust, and moldy grains, further contributing to the deterioration of damaged stored products [[22], [23], [24]].

**Fig. 1:**
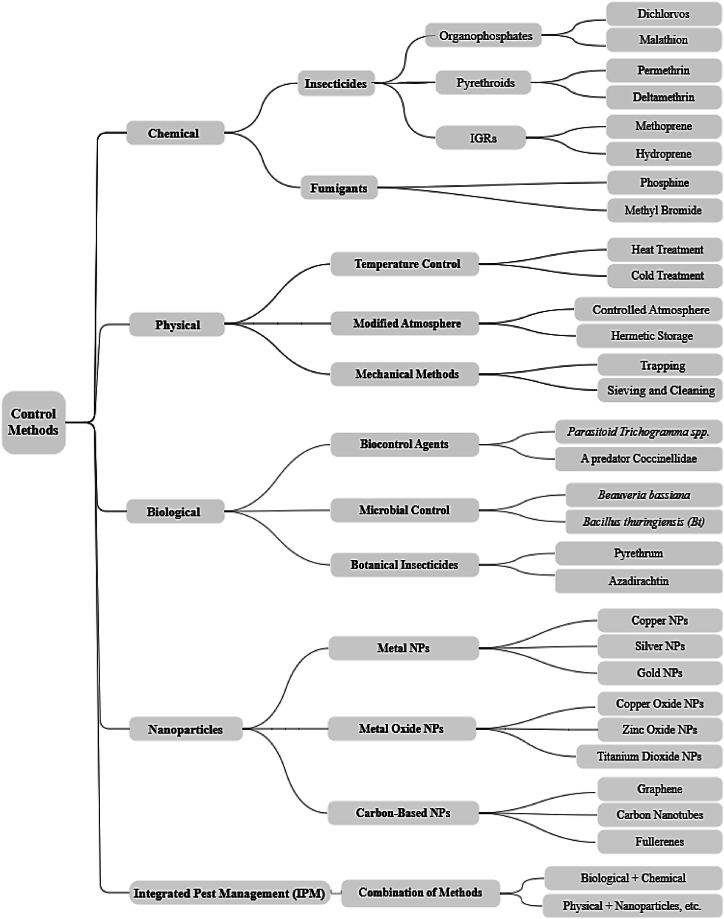
A schematic representation for pest control strategies, along with Integrated Pest Management (IPM).

Effective pest management strategies have been a focal point of research across continents for several decades. These strategies can be broadly categorized into physical, chemical, and biological methods (Fig. 1), each with its own advantages and limitations. Biological control approaches, widely regarded as the most effective and safest pest management strategies, employ biological agents such as natural predators, parasitoids, and microbial agents for pest control. For instance, parasitoid *Trichogramma* spp [25]. and predatory beetles (Coccinellidae) [26] are used effectively in these strategies. Microbial controls using *Bacillus thuringiensis* (Bt) and *Beauveria bassiana* [26], botanical insecticides like neem extracts (Azadirachtin) and pyrethrum [27], and pheromones or semiochemicals [28] have also been studied for their natural pest-repellent properties, mass trapping lures, and mating disruption effects. These control measures are suggested to deter feeding and oviposition, providing long-term, self-sustaining, and eco-friendly pest management without the drawbacks associated with chemical treatments [29].

Physical methods for managing stored product pests contain temperature control [30], modified atmospheres [31], and mechanical techniques [32]. Temperature control includes both heat and cold treatment. Extreme temperatures, whether from heating or cooling, directly impact insects and mites, making their survival and reproduction less feasible [24]. Similarly, unregulated or suboptimal humidity levels can restrict the development and proliferation of stored product pests [33]. Modified atmospheres, achieved through hermetic storage and controlled conditions, create unfavorable environments for pests. Mechanical methods involve sieving, cleaning, and trapping to physically remove or isolate pests from stored products. These physical measures—such as mechanical methods, temperature control, and humidity control—have effectively disrupted the life cycle of stored product pests [30,31].

Chemical insecticides and fumigants for pest control include various compounds, including organophosphates (e.g., dichlorvos and malathion) [34], pyrethroids (e.g., permethrin and deltamethrin) [35], and insect growth regulators (IGRs) like methoprene and hydroprene [36]. Chemical methods have been widely used and favored for decades in managing storage pests. However, the indiscriminate use of chemical insecticides and the frequent development of resistance among insect pests make these methods less reliable [37]. These chemicals also pose risks to human health, contribute to environmental contamination, and negatively affect the quality, germinability, and viability of grains and seeds [38,39]. Notable insecticide resistance has been reported among *R. dominica, Sitophilus oryzae, Cryptolestes ferrugineus,* and *T. granarium* [12,40]. On the other hand, there is a continuous effort to reduce the usage and risk of chemical pesticides by 50 % by 2030, as well as to halve the use of harmful pesticides [41].

Other physical methods listed below also have some limitations. For example, although effective in disrupting the metabolic activity of insects and eventually causing death through reduced oxygen (O_2_) concentration [42], finding economically feasible hermetic storage facilities remains a challenge. Similarly, methods such as low-pressure storage, modified atmosphere storage, ozone treatment, thermal treatment, irradiation, and other techniques are either impractical for large-scale implementation, raise significant safety concerns regarding human exposure, or yield suboptimal results unless integrated with other pest management approaches [14]. Recent studies have suggested some kind of NPs in various forms, including a nanoformulation (an NPs-based formulation for enhanced delivery) [43], a nanoemulsion (a dispersion of NPs in liquids), or a nanosuspension (a colloidal suspension of NPs) [44], as emerging and promising alternatives for managing stored-product insect pests [13]. The use of NPs has the potential to overcome the constraints associated with the conventional pesticides by boosting insecticide action, improving the stability of active components, lowering the dose of the required insecticide, and conserving agronomic inputs [19,20,45].

### 2.1 Greener approach to NPs

**Fig. 2:**
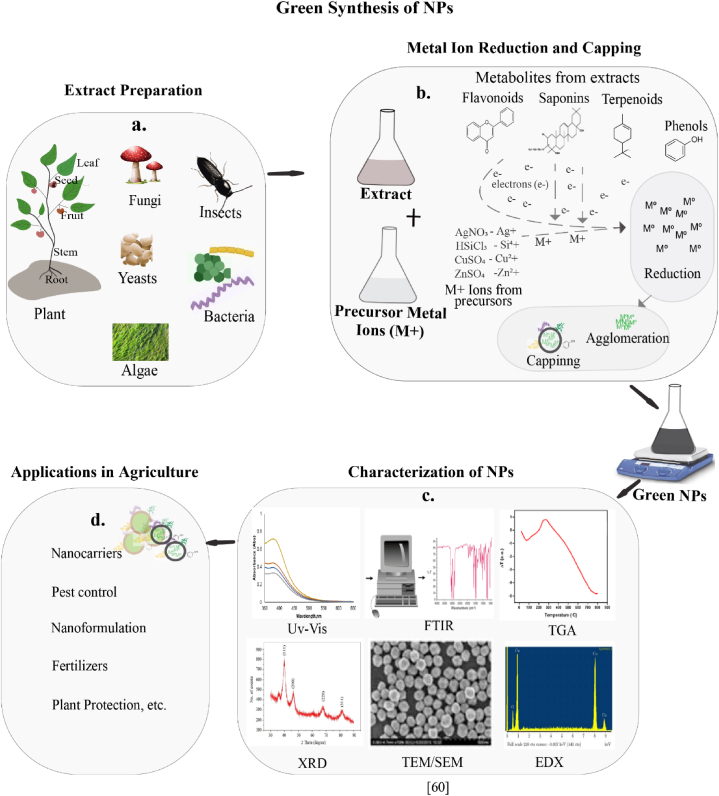
**Schematic illustration of the various stages of green synthesis and characterization of NPs.** a) Preparation of extracts from various sources (plants, bacteria, algae, fungi, yeasts, insects), b) Reduction and capping of metal ions using metabolites in the extracts, c) Characterization of NPs with some techniques, d) Application of NPs in diverse areas.

Over the past few years, the current research interests, advancements and achievements in the area of pest management have been driven by NPs [19,46]. In the synthesis of NPs, bulk materials or ions are disrupted or reduced and nucleated to form particles ranging from 1 to 100 nm in size, using specialized physical, chemical, or biological methods [47,48]. These particles are termed 'nanoparticles,' derived from the Greek word for 'dwarf,' to represent one billionth (1x10^^- 9^) [49]. Among the NPs preparation methods, physical approaches are the expensive since they require sophisticated equipment, trained operators, regulated conditions, and high supplies of energy [50]. Chemical approaches have the limitations of using expensive, dangerous and environmentally toxic chemicals, too [51]. Besides, researchers have been exploring the biological approaches referred to as green methods for the preparation of NPs. The formation of green NPs typically occurs in three stages: reduction of metal ions to their monovalent state, nucleation of reduced metals, and capping of atomic clusters [49,51]. A visual clarification of the green synthesis process, including the preparation of extracts from various sources, the reduction and capping of metal ions, characterization of the resulting NPs, and their potential applications in agriculture is presented in Fig. 2.

The biological, or namely green, method uses biologically-origin extracts from living organisms such as insects [52], plants [53], bacteria [54], yeast [55], algae [56], and fungi [57] for the synthesis of NPs. These materials have been extensively explored, and many studies have achieved successful outcomes [[52], [53], [54]]. Among the biological agents, plants and their extracts are the most preferred materials due to their abundant content of primary and secondary metabolites such as flavonoids, saponins, terpenoids, phenolic compounds, polysaccharides, proteins, and amino acids that are important for the reduction and capping of NPs in the synthesis [58,59]. In addition, the use of plant extracts for the synthesis of NPs minimizes the production costs by eliminating the need for special culturing and isolation processes, and characterization of microorganisms [60,61]. In green synthesis, parameters such as the metal precursor and extract concentration, pH value, reaction time, reaction temperature, and plant type must be optimized for controlled NPs synthesis [62]. The plant type is particularly important, as a high content of plant extract, required for the efficient reduction and stabilization of NPs, plays a crucial role in determining their size and morphology [54]. Various analytical techniques, including X-ray Diffraction (XRD), Fourier- Transform Infrared Spectroscopy (FTIR), Transmission Electron Microscopy (TEM), Ultraviolet–Visible Spectroscopy (UV–Vis), Thermal Gravimetric Analysis (TGA) and Surface charge measurements (SCM) are employed to verify the successful synthesis of the NPs and elucidate their physicochemical properties [63,64].

NPs prepared by green methods have been extensively studied for applications in crop production, nanofertilizers, nanosensors, nanopesticides, and soil quality improvement. Many of the studies, as summarized by [65] have yielded remarkable results. For instance, NPs of copper, gold, silver, zinc and other metallic NPs have been suggested to significantly enhance the current agriculture system [66,67]. Enhanced wheat germination, growth, grain yield, and biomass, along with reduced toxicity compared to the non-metal forms of NPs, were observed with the application of zinc oxide NPs [68,69]. Thus, it is believed that NPs could potentially replace conventional chemical pesticides, which are environmentally toxic and pose risks to humans and other aquatic and terrestrial life forms.

### 2.2 NPs against storage pests

Studies on potential applications of NPs in insect pest control are a rapidly evolving field; with ongoing research focused on optimizing NP formulations, their delivery mechanisms, and environmental compatibility [70,71]. Several types of metallic NPs and their oxide counterparts, including alumina (AlNPs), copper (CuNPs), copper oxide (CuONPs), gold (AuNPs), graphene oxide (GO), potassium (KNPs), selenium (Se NPs), silver (Ag NPs), silica (SNPs), silicon dioxide (SiO2 NPs), zinc oxide (ZnO NPs), and inorganic NPs such as polymeric and chitosan NPs, have been explored for their insecticidal properties (Table 1). According to reports, NPs offer numerous advantages over conventional insecticides, including enhanced chemical solubility, lower dosage requirements, and controlled release of pesticide ingredients [17]. These advantages could mitigate the unfavorable side effects associated with conventional and phased-out chemical insecticides [19]. Furthermore, NPs can be utilized as insecticide delivery agents and enhancers of nutrient and water absorption [72]. Another potential application of NPs is their utilization as nanofertilizers for soil nutrient improvement, where nanoforms of macro and micro plant nutrients, such as nitrogen, phosphorus, and potassium, are supplied to crops in a regulated manner [73,74]. In nanofertilizers, nutrients are bound to nano-dimensional structures, either alone or in combination, resulting in slower nutrient release compared to standard fertilizers [74,75]. Nanofertilizers derived from certain nanoformulations offer advantages such as increased chemical solubility, improved stability, enhanced mobility, slow release, and improved insecticidal activity [65,73]. These nanoformulations—nanoscale preparations to enhance the delivery, efficacy, and stability of active agents—are free of hazardous organic solvents, avoiding the expense, flammability, and toxicity of conventional emulsifiable pesticide formulations. Additionally, nanopesticides—pesticides formulated with NPs for gradual release—require fewer applications, addressing the challenges of conventional insecticides, which often require repeated use and contain unhealthy formulations [76,77]. The high surface area of NPs makes them effective nanocarriers for loading large amounts of pesticides with quick delivery potential to the target [78]. Thus, NPs could help improve soil health, sustain the geobiological soil cycle by enhancing food crop nutrition, and also reduce farmers' fertilizer expenses by enabling site-specific and controlled release of active substances in agricultural inputs [79,80]. Integration of nanotechnology in pest management could revolutionize the agricultural industry, lead to improved crop yields and reduce the infrequent application of harmful chemical pesticides [20]. As highlighted earlier, it is possible to suggest that NPs in pest control could represent a promising and environmentally friendly alternative to conventional pesticides in sustainable pest management strategies with numerous advantages.

**Table 1:** Nanopesticides against primary and secondary storage pests.

	Type of NPs	Characteristics of NPs	Target insect	Lethal Concentration (LC 50/90), Lethal Time or Key findings	Reference
1	S NPs	Rice straw-based green S NPs, spherical, ∼4 nm in size	*Callosobruchus maculatus*	− 100 % mortality at 200 ppm after 48 h of exposure - No adverse effect on cowpea seed germination; instead, enhanced (2 %) germination efficiency was recorded	[81]
2	Ag, Se, SiO_2_, CuO, TiO_2_, and ZnO NPs	Fungus *Fusarium solani* based NPs, 8–33 nm in size	*Callosobruchus chinensis* and *C. maculatus*	- SeNPs and TiO_2_ NPs reduced *C. chinensis* egg hatchability by 22.8 % and 17.7 %, respectively - SeNPs reduced egg-to-adult survival by 18 % - TiO_2_ NPs reduced larva-to-adult survival by 11 % and egg-to-adult survival by 15 % in *C. maculatus*	[82]
3	Ag, and Cu NPs	The NPs were spherical, crystalline, and 54.1 nm (Ag NPs) and 74.9 (Cu NPs) in size.	*C. chinensis*	- Both NPs at 1000 ppm resulted in 100 % mortality of pulse beetles after one month - Enhanced seed germination and seed quality	[16]
4	Bt-ZnO NPs	*Bacillus thuringiensis-*derived, hexagonal wurtzite, 20 nm, zeta potential: −12.7 mV	*C. maculatus*	- Reduced fecundity and hatchability - Reduced mid-gut enzyme activity − 100 % mortality at 25 μg/mL - The LC50 value was 10.71 μg/mL.	[107]
5	NiO NPs	Green NiO NPs, porous, 9–16 nm	*C. maculatus*	- Reduced fecundity and extended developmental period at 40 ppm - Significant impact on seed germination at 40 ppm	[83]
6	ZnO NPs Nano silica	No details provided	*C. chinensis*	− 100 % mortality with nano silica and nano zinc oxide at 1000 ppm on the 5th and 7th day, respectively - No seed damage at 1000 and 750 ppm even after 6 months; nano silica outperformed nano zinc oxide by 1.3 %	[108]
7	TiO_2_ NPs: Anatase, aeroxide p25, degussa p25	Spherical, crystalline, 21–49 nm	*C. chinensis*	- Improved Nonabokra rice germination up to 100 % mortality of bean weevils at 1–2.5 g/kg doses of Aeroxide P25 treatment after 14 days - Limited antibacterial activity and variable cytotoxicity to brine shrimp	[109]
8	Nano zeolite	Inert dust- powder, Light gray, 40–45 nm, sheet-like particles	*Tribolium confusum* *C. maculatus*	− 96.66 % mortality for *T. confusum* at 800 ppm after 2 weeks, 100 % mortality for *C. maculatus* at 500 ppm after 3 days - Reduced adult progeny production and egg laying - SEM showed dehydration due to cuticle scratching and leg damage	[103]
9	GO powders	Nano- and micro-sized, structural and chemical variability	*Sitophilus oryzae, R. dominica, Prostephanus truncates*	- Nano-graphene at 1000 ppm caused faster mortality via cuticle disruption and respiratory interference. GO had no efficacy.	[110]
10	Co_3_O_4_-NPs	41 nm, eco-friendly synthesis using Nodosilinea nodulosa	*S. oryzae, T. castaneum, R. dominica*	- Ineffective as an insecticide; displayed dose-dependent phytotoxicity.	[111]
11	Non-biogenic and biogenic ZnO NPs	Sol–gel method synthesis, 20–30 nm	*S. oryzae*	- Non-biogenic ZnO NPs effective against *S. oryzae* at LD50 values of 22.33 mg/100 g - Promoted maize seed growth	[112]
12	ZnO NPs SiO_2_ NPs	SiO_2_ <100 nm, spherical; ZnO: 12–21 nm, spherical	*S. oryzae, T. castaneum, C. maculatus*	- Mortality: 81.6 % for *C. maculatus* at 0.3 g/kg SiO_2_ NPs, 98.3 % for S. oryzae at 2 g/kg, 58.3 % for *T. castaneum* at 8 g/kg - Toxic to *S. oryzae* and *C. maculatus* - Histopathological examination: liver abnormalities observed, but normal lung condition in treated animals	[113]
13	Pure Ag NPs, Green Ag NPs, Leaf extract	Pure Ag NPs: 0–30 nm, Green Ag NPs: 52 nm	*T. castaneum,* and *C. maculatus.*	- Higher larvicidal effect for Green-Ag NPs with LC50 values of 2.019 for *T. castaneum* and 3.366 for *C. maculatus* - Nerium oleander leaf extract had lower larvicidal effect	[86]
14	TiO_2_ NPs	Spherical, 10–30 nm diameter, average size 74.8 ± 0.649	*S. oryzae*	− 85 % mortality at 100 % concentration after 120 h - Reduced total soluble proteins, improved nutritional content, increased catalase activity with higher NP concentration	[87]
15	Ag NPs	Spherical, 40.56 nm, zeta potential: −27 mV, UV–Vis peak: 450 nm	*S. oryzae, T. castaneum*	- LC50: 6.08 μg/mL for *S. oryzae*, 7.03 μg/mL for T. castaneum − 100 % mortality at 0.1–0.2 g/100 g seeds for *S. oryzae* after 2 weeks − 1%DP showed increasing mortality rates for both *T. castaneum* and *S. oryzae* with higher concentrations and exposure intervals	[88]
16	PNs (Polymeric nanoparticles)	NPs were Loaded with *Origanum vulgare* and *Laurus nobilis* essential oils, 295–429 nm, 90 % loading efficiency, polydispersity index ≤0.25	*S. oryzae, Lasioderma serricorne*	- Altered nutritional physiology and behavior, enhanced insecticidal effects of essential oils - Minimal toxicity to non-target aquatic organisms (*Artemia salina*) and primary osteoblast cells. - More effective when combined with β-cypermethrin	[84]
17	ZnO NPs	No details provided	*S. oryzae*, *S. cerealella*	- Significant insecticidal activity depending on concentration and exposure time − 150 ppm resulted in 88.89 ± 11.11 % mortality after 14 days on wheat grains	[93]
18	AgNO_3_ NPs	No details provided	*S. granarius*	− 46.2 % mortality at higher concentration (500 ppm) after 72 h − 36.2 % repellency after 36 h	[89]
19	CuONPs	Synthesized after 24 h at 30 °C, 150 rpm, spherical, crystallographic, 14.0–47.4 nm	*S. granarius*, *Rhyzopertha dominica*	- Mortality rates: 55–94.4 % for *S. granarius*, 70–90 % for *R. dominica* - LC90 = 300 mg/100 g seed after 8–10 days	[18]
20	CS/PO NPs	Zeta potential: −21.12 mV, average diameter <563.3 nm, UV–Vis peak: 227 nm	*T. castaneum*, *S. oryzae*	- Peppermint oil-encapsulated CS NPs showed high insecticidal efficacy - LC50: 34.79 μL/L for *T. castaneum*, 28.61 μL/L for *S. oryzae* − 100 % mortality at 75.0 μL/L	[85]
21	ZnO NPs, SiO_2_	Sourced from Nanotech Company: no details provided	*S. oryzae, C. maculates, T. castaneum*	- ZnO: 100 % mortality in *S. oryzae*, 88.3 % in *C. maculatus*, 36 % in *T. castaneum* at highest concentrations. - SiO_2_: 98.3 % in *S. oryzae*, 98 % in *C. maculatus*, 57 % in T. castaneum at the highest concentrations.	[113]
22	SiO_2_-NPs	Spherical, <50 nm, highly negative zeta potential (−40 mV)	*S. oryzae, T. castaneum, R. dominica, Orizaephilus surinamenisis*	- LC50: after 24 h: 1.240 g/kg for *S. oryzae*, 1.450 g/kg for *T. castaneum*, 0.336 g/kg for *R. dominica*, 0.768 g/kg for *O. surinamenisis*. - NPs applied to a soil at 10 g/kg improved maize growth and yield - Combining 50 % NPK with 10 g/kg SiO_2_-NPs enhanced agronomic traits the most.	[74]
23	ANP, ZNP, TNP	Commercially procured, ANP: <45 nm, TNP: 45–60 nm, ZNP: 50–350 nm.	*S. oryzae*	- High effectiveness for all NPs against *S. oryzae* - Insecticidal activity at the lethal Dose 2 g/kg: aluminium oxide (ANP) significant by day 4; titanium dioxide (TNP) and zinc oxide (ZNP) moderate by day 14.	[92]
24	NSA (Nanostructured Alumina)	Bi-modal size distribution: 1.5 μm-350 nm, platelet morphology, thickness: 45 nm	*S. oryzae*	- Parental survival: 36 % after 7 days, 13 % after 14 days, 0 % after 21 days at 250 ppm - Reduced grain weight loss and frass production	[114]
25	Ag NPs	Rod-shaped, 25–80 nm	*S. oryzae*	− 100 % mortality after 7 days - LD90: 168.28 ± 15.38.	[90]
26	Nanodusts	Highly porous, 16.76–74.42 m^2^/g surface area, ∼9.54 nm	*S. oryzae, R. dominica*	− 100 % mortality for *S. oryzae*, significant mortality for R. dominica after 9 days of continuous exposure - Higher efficacy at 43 % RH, reduced efficacy at 75 % RH	[91]
27	Ag NPs	Irregular, 15–65 nm, zeta potential: −28.9 mV	*R. dominica* *T. castaneum, S. oryzae*	- Mortality at 150 μg highest concentration): 47.4 ± 0.16 % for *S. oryzae*, 52.8 ± 0.24 % for *R. dominica*, 55.2 % for *T. castaneum*	[92]
28	Nanostructured alumina (NSA)	Platelet morphology, 45 nm	*R. dominica, S. oryzae*	- Lethal time (LT95) for *S. oryzae*: 10.9 days at high humidity, 11.66 days at medium, 10.6 days at low - Lethal time (LT95) for *R. dominica*: 25.04 days at high humidity, 7.26 days at medium, 2.64 days at low	[97]
29	Aerosil 200 NPs, Chemical Silica NPs, Bio-Silica NPs	Aerosil 200: Commercially obtained; Chemical Silica: Lab-prepared; Bio-Silica: Rice husk derived.	*R. dominica* *C. maculatus*, *T. confusum*	- LC50 for *R. dominica* on the 3rd day: Chemical Silica: 0.19 g/kg grain, Aerosil 200: 0.23 g/kg grain, and Bio-Silica: 4.02 g/kg grain	[100]
30	Ag NPs	Alkaloids of *Peganum harmala* derived, 22.5–66.2 nm	*T. granarium*	- LC50: 4.7–11.4 μg/cm^2^ for larvae, 4.1–10.2 μg/cm^2^ for adults - Significant growth impairment in second instar larvae at sublethal concentrations - High percentage of malformed larvae and pupae	[99]
31	SNPs, ANPs	Commercially purchased	*T. granarium*	− 100 % mortality of second instars. - Reduced fecundity and production of significantly fewer eggs compared to control	[98]
32	CNTs	Multiwalled, 50–60 nm, derived from Iraqi date palm seeds	*T. granarium*	− 70–100 % adult mortality, 46–88 % larval mortality after 8 days at 25, 50, 100 ppm - Significant effect under laboratory conditions	[101]
33	CuNPs	Bacteria extract-derived, spherical, crystalline, 10–70 nm, zeta potential: −26.00 mV	*T. castaneum*	- Significant toxicity against *T. castaneum* - LC50: 36.89 μg/mL after 5 days	[54]
34	CuNPs	Fungal-mediated, green-synthesized, spherical, crystalline, 15.67–62.56 nm.	*Tenebrio molitor*	- Effective against *T. molitor* - The LC50 and LC90 values for *T. molitor* were 6.487–29.363 μg/mL, respectively	[115]
35	CuNPs	Plant-derived, crystalline, poly-dispersed, 0.47–25.58 nm	*T. molitor*	- The LC50: 338.680 μg/mL	[116]
36	SNPs	Amorphous, lipophilic, 15–20 nm	*T. castaneum*	− 57 % mortality at 0.5 mg/cm^2^, 73 % at 1 mg/cm^2^, and 96 % at 1.5 mg/cm^2^ after 7 days of treatment.	[117]
37	Polymeric NPs (Chitosan-based)	Garlic EO (231.14 nm) and cinnamon EO (303.46 nm), high stability, encapsulation efficiency >76 %	*T. castaneum*	- Enhanced repellency (66.66 %) and anti-nutritional effects at LC30, with significant reductions in growth, consumption rate, and feeding deterrence.	[118]
38	CuNPs	*A.cornigera* derived CuNPs: 63–153 nm, zeta potential: 9.6 mV; *A. purpurea* CuNPs: 87–193 nm, zeta potential: −32.7 mV	*T. castaneum*	− 90 % mortality for *A. cornigera*-derived CuNPs, 70 % for *A. purpurea*-derived CuNPs	[46]
39	Bt-Ag_2_ O NPs	18.24 nm, stable, well-dispersed, highly crystalline	*T. castaneum*	- LC50 value of 0.06 % on day 14 for *Bacillus thuringiensis*-derived Ag NPs against *T. castaneum*	[119]
40	Clove oil NPs	PEG NPs, 179 ± 1.69 nm, irregular shape, 77 % encapsulation efficiency	*T. castaneum*	- Maintained over 70 % mortality at 14.6 % concentration and 90 % mortality at 15.2 % concentration for 16 weeks - Demonstrated sustained contact toxicity	[105]
41	Ag NPs	18.24 nm, stable, well-dispersed, highly crystalline	*T. castaneum*	- Ag NPs combined with malathion at 50 mg resulted in 100 % mortality after 9 days - Reduced oviposition and feeding	[37]
42	CS/PNO NPs	Spherical, 527.5 nm, high oil loading efficiency, zeta potential: −5.34 m	*S. oryzae*, *T. castaneum*	- LC50 values: 25.03 ppm for *S. oryzae*, 29.02 ppm for *T. castaneum* - Reduced vitality, feeding, and acetylcholinesterase activity in larvae - Mortality increased with NP concentration; Si NPs more toxic than Ag NPs	[85]
43	Ag NPs, Si NPs	Ag NPs: 40 nm, zeta potential: −19 mV; Si NPs: 35 nm, zeta potential: −19 mV	*T. castaneum*	- Si NPs induced 70 % mortality, Ag NPs 40 % mortality - LC50 values: 438.3 ppm for Si NPs, 657.4 ppm for Ag NPs - Mortality rates increased with both NPs concentrations; however, Si NPs was more toxic to the pest.	[120]
44	Ag NPs	Green-synthesized, crystalline, spherical, 50 nm	*T. confusum*	- Mortality rates of 83-77 % using filter-paper residue and feeding methods - LC50: 30.62 ppm, LT50: 9.92 days in filter-paper residue tests.	[121]

#### 2.2.1 NPs against primary storages pests

**NPs against the pulse beetle, *Callosobruchus maculatus/chinensis* (Coleoptera: Bruchidae):** Alongside various types of NPs (see Table 1), a study on the potential of rice straw-derived SNPs in managing *C. maculatus* infestations in stored grains not only highlights promising pest control strategies but also demonstrates environmentally friendly NP synthesis methods that could reduce environmental waste [81]. A recent study has provided clearer insights into species- and developmental stage-specific effects of various NPs, such as Ag, Se, SiO_2_, CuO, TiO_2_, and ZnO NPs [82]. The study reported that different species of *Callosobruchus pests*, including *C. chinensis* and *C. maculatus*, have exhibited different level of susceptibility to NPs. Eggs were more susceptible than larvae. Specifically, Se and TiO_2_ NPs reduced *C. chinensis* egg hatchability by 23 % and 18 %, respectively. SeNPs led to an 18 % reduction in egg-to-adult survival, while TiO_2_ NPs reduced larva-to-adult survivorship by 11 % and egg-to-adult survival by 15 % in *C. maculatus*. Additionally, the smaller size of *C. chinensis* eggs (23 % smaller than *C. maculatus*) was found to be associated with higher mortality resulted from NP exposure. It is concluded that biosynthesized SeNPs and TiO_2_ NPs could have potential for controlling stored bean pests. The comprehensive study by Ref. [16] indicated that green NPs such as Ag NPs and CuNPs did not adversely affect soil microbial organisms or seed quality. In fact, these NPs enhanced seed quality parameters, with Ag NPs at 1000 ppm increasing germination by 81.67 % and CuNPs at 1000 ppm by 79.33 %, and achieved 100 % mortality of the tested pest after one month. They also promoted superior root growth and enhanced biochemical activities of alpha amylase and catalase enzymes. While elevated enzyme activities in their study could suggest potential abnormalities, the authors interpreted these findings positively, likely because the enzyme activity was not associated with increased production of reactive oxygen species (ROS). Another study, conducted by Ref. [83], demonstrated a significant reduction in seed germination by 79.12 % at 40 ppm concentration of NiO NPs treatment. Additionally, the study observed decreased fecundity and an extended developmental period in a dose-dependent manner. This finding suggests that NPs at higher concentration can produce notably varied outcomes, and caution should be exercised in their application or consideration for application. Furthermore, all these applications not only offer promising pest control strategies but also demonstrate environmentally friendly NP synthesis methods that could reduce environmental waste.

**NPs against *Sitophilus weevils* (Coleoptera: Curculionidae):** Pest species under the genus *Sitophilus*, particularly, the rice weevil (*S. oryzae*) and the granary weevil (*Sitophilus granarius*), are well-known for their potential to cause huge post-harvest losses. NPs synthesized using polymers [84], chitosan [85], SiO_2_ NPs [74], and metallic NPs such as Ag NPs [86], copper oxide (CuO) NPs [18], and titanium dioxide (TiO_2_) NPs [87] have been studied against these pest species (Table 1). These studies present a case that NPs could be an efficient and environmentally harmless substitute for chemical pesticide control against rice and maize weevil pests. For instance, the study by Ref. [18] found that CuO NPs were effective in controlling *S. granarius* and *R. dominica*, with additional benefits to wheat growth and morphology. Another study on the efficacy of *Juniperus phoenicea* (JP)-based JP- TiO_2_ NPs found that these NPs were effective against the rice weevil, while also improving the nutritional value of treated grains, including fats, ash, crude fiber, and total carbohydrates [87]. While these NPs are promising, it is noteworthy that their application should be accompanied by proper safety measures, as histopathological examination in the above study revealed some hepatic changes in treated male mice. Other studies on various NPs, such as Ag NPs [[88], [89], [90]], nanoalumina dusts [91], ANPs, TiO_2_ NPs, and ZnO NPs [92,93], SiNPs and Ag NPs [94], and chitosan NPs [85], suggest that pests of rice and grain weevils could potentially be controlled using various kinds of NPs. However, standardized comparative studies are necessary to clarify the varied efficacies and modes of action of these NPs against these pests.

**NPs against lesser grain borer, *Rhyzopertha dominica* (F.) (Coleoptera: Bostrychidae):** Numerous studies have demonstrated the insecticidal properties of NPs against *R. dominica*, a significant pest of stored grains. Ag NPs, synthesized through a phytoreduction method using the salt marsh grass *Myriostachya wightiana*, showed insecticidal efficacy against *R. dominica* and other pests of stored grains such as *T. castaneum,* and *S. oryzae* [95]. The biosynthesized Ag NPs demonstrated insecticidal activity against these pests, with mortality percentages recorded after various post-treatment time periods. In another study, silica NPs, specifically Aerosil and Nanosav Si NPs, applied on wheat and peeled barley, demonstrated insecticidal efficacy against *R. dominica* and *Tribolium confusum* adults [96]. Green CuO-NPs exhibited time-, dosage-, and size-dependent insecticidal activity against the two wheat grain pests, *R. dominica* and *S. oryzae* [18]. A study on nanostructured alumina (NSA) found that this NP could be a promising insecticide against *S. oryzae* and *R. dominica* even at lower concentrations [97]. The utilization of diverse biological sources such as *Myriostachya wightiana* for synthesizing and applying various NPs against insect pests implies the innovative strategies being explored in nanotechnology for pest control. However, existing studies on the long-term effects of NPs use on non-target organisms and ecosystems are limited, especially concerning these pests, and further research is essential despite the promising results. Similarly, as with other stored product pests, scalability studies are limited. Research is necessary to ensure that these methods can be practically implemented in large-scale agricultural settings to effectively control *R. dominica* and other pests.

**NPs against the Khapra beetle, *Trogoderma granarium* (Everts) (Coleoptera: Dermestidae):** The evaluation of SNPs and ANPs application on *T. granarium* resulted in more than 90 % mortality of second-instar larvae at a concentration of 200 mg of NPs per 500 g of various stored products, such as barley, rice, wheat, white maize and yellow maize [98]. Different stages exhibited varying susceptibility, with earlier larval instars being more affected than later stages and adults. Exposure to NPs also reduced the fecundity of adults and resulted in significantly fewer eggs compared to controls [99]. The differential susceptibility observed across various life stages of the khapra beetle indicates the importance of adjusting and targeting specific stages in its life cycle to maximize efficacy in pest control. Similarly, a study on the effectiveness of Ag NPs synthesized using harmala alkaloids extracted from the plant *Peganum harmala* as an insecticide revealed that these NPs exhibited significant insecticidal and growth-inhibitory effects on the khapra beetle [99]. The substantial efficacy of SNPs, ANPs Ag NPs, Silica NPs fumed silica (Aerosil 200) [100], and carbon nanotubes (CNTs) [101] against *T. granarium* suggests promising potential for their application in pest control. However, these results should be weighed against potential environmental and health impacts. Further research on long-term effects, economic feasibility, and regulatory approval is necessary to ensure the safe and effective real-world application of various types of NPs.

#### 2.2.2 NPs against secondary storages pests

The preservation of agricultural commodities during storage, which faces significant challenges from secondary storage pests, is crucial for ensuring food security and minimizing post-harvest losses. Losses due to insect pests are estimated to range from 9 % in developed countries to 20 % or more in developing countries [102]. Some of the major secondary storage pests that damage stored products include *T. castaneum* (Herbst) (Coleoptera: Tenebrionidae), commonly known as the red flour beetle; *Tenebrio molitor* (Coleoptera: Tenebrionidae), commonly known as the yellow mealworm; the potato tuber moth *Phthorimaea operculella*, and the rice moth *Corcyra cephalonica*. Nanoparticle-based insecticides, with their range of innovative formulations and synthesis methods, represent a promising approach to controlling *Tribolium* and other secondary storage pests. *T. castaneum* is a major pest in stored grain products. Research by Ref. [85] demonstrated that use of chitosan NPs loaded with *Piper nigrum* and peppermint essential oils, which enhanced the controlled release of the NPs, was effective for sustained pest control against *T. castaneum* and *Sitophilus oryzae*. In another study, *C. maculatus* and another *Tribolium* species *Tribolium confusum* were effectively targeted using nano zeolite as an alternative pest control agent [103]. Eco-friendly synthesized copper nanoparticles (CuNPs) from bacterial extracts [54]*, Acacia cornigera* and *Annona purpurea* [46] were also found to be effective against *T. castaneum*. Additionally, the efficacy of green silver nanoparticles (Ag NPs) synthesized using *Lemna minor* biomass displayed long-term impact on both *T. castaneum* and *S. oryzae* [88]. While various types of NPs have been found effective against pests on their own, there is also potential to combine these nanomaterials with other insecticides in pest control strategies. It has been demonstrated that Ag NPs combined with malathion, an organophosphate insecticide*,* exhibited synergistic effects, resulting in higher mortality rates and repellency against *T. castaneum* compared to using them individually [37]. The combination of Cu/CuO-NPs with deltamethrin also significantly enhanced the effectiveness of the insecticide against *Bactrocera oleae* adults. Although Cu-NPs alone have caused higher adult mortality and reduced the number of stings, pupae, and female offspring compared to the recommended doses of deltamethrin, the combination of Cu-NPs with deltamethrin significantly reduced the total number of offspring at a higher level than that achieved with deltamethrin alone [104].

NPs also have another potential application, as they can release chemical substances in a controlled and regulated manner. Study by Ref. [105] demonstrated that polyethylene glycol (PEG) NPs loaded with clove essential oil provided sustained release and protection against oil degradation over 16 weeks. While protective effect against oil degradation is noteworthy, further optimization is needed to boost insecticidal efficacy. Integrating NPs with other chemical insecticides also necessitates careful consideration of environmental and health impacts. For instance, malathion is considered harmful due to its chronic effects, which include neurological and reproductive issues, genetic disorders, carcinogenicity, and endocrine disruption [106]. These studies collectively highlight the promise and challenges of nanoparticle-based insecticides and emphasize the need for continued research to optimize formulations and ensure practical, sustainable pest control solutions. With further optimization, these nanoparticle-based strategies have the potential to offer prolonged efficacy and enhanced insecticidal effectiveness, and pave the way for more effective and environmentally responsible pest management solutions.

## 3 Cellular, biochemical and molecular studies as NPs’ mode of action

Early studies on nanopesticides primarily focused on examining toxicity and immediate mortality rates resulting from NP exposure. As a result, although standardization—ensuring consistent methods and results across studies—remains an apparent issue, extensive information on their effects against primary and secondary storage pests, as well as insect vectors, is available. Additionally, despite compelling evidence supporting the potential of NPs as alternatives to conventional chemical insecticides, a detailed understanding of their cellular, molecular, and biochemical impacts on insects remains limited, particularly for storage pests [76,122,123]. However, recent studies have expanded to examine the cellular, physiological, molecular, and biochemical effects of NPs exposure in insects. These investigations have increasingly uncovered how oxidative stress, cellular homeostasis disruption, and other mechanisms interact synergistically, suggesting their collective impact on pest physiology. This has provided more detailed insights into the mechanisms of NP actions on both target and non-target organisms, as discussed in the following section.

### 3.1 Cellular, biochemical and molecular modifications

**Fig. 3:**
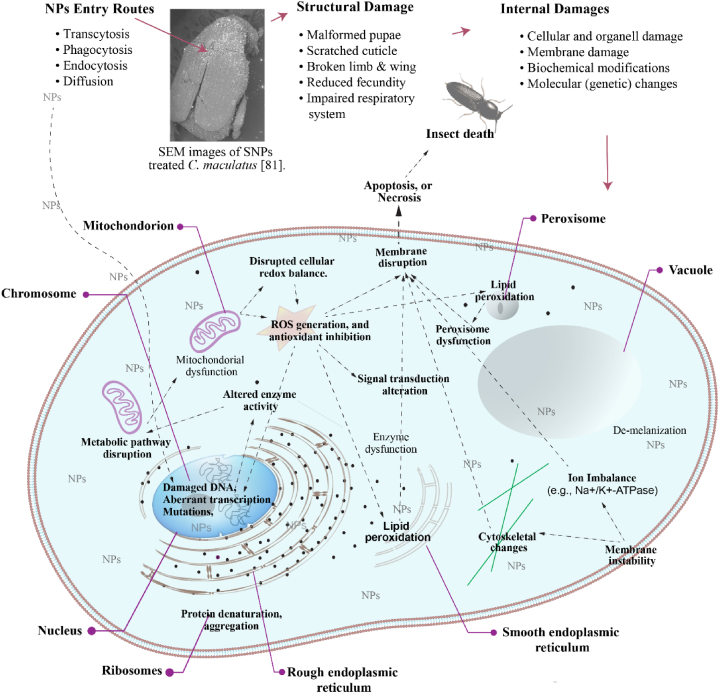
**NPs-Induced Physical and Cellular Disruptions in Insects**. The *Figure* illustrates how NPs disrupt insect physiology, from entry routes (e.g., endocytosis, diffusion) to structural damage (e.g., malformed pupae, scratched cuticle, broken legs and wings) and cellular dysfunction (e.g., oxidative stress, membrane instability), leading to insect death.

Evidence indicates that the insecticidal properties and mechanisms of NP actions are complex and involve numerous physical, as well as biochemical and molecular modifications in the cell (Fig. 3). NPs induce significant morphological damage; for instance, large amounts of silica NPs accumulate between the joints of the thorax and abdomen, resulting in physical damage such as the loss of legs [81]. Insects exposed to nano zeolite exhibited broken legs and wings [103], while nickel oxide NPs caused significant structural changes in the testicular tissues, severe damage to subcellular organelles, and deformities in the spermatozoa heads and flagella of the ground beetle *Blaps polycresta* [124]. Similarly, aluminum oxide (Al₂O₃) NPs induced significant testicular abnormalities in migratory locusts (*Locusta migratoria*) due to aluminum accumulation in tissues, leading to DNA damage, oxidative stress from ROS generation, and increased apoptosis [125]. Treatment of first instar mosquito *Culex pipiens* larvae with a sublethal concentration (LC20, 0.24 g/L) of ZnO NPs for 72 h, followed by a 72-h recovery period, resulted in significant zinc accumulation, pathological changes in midgut ultrastructure, and a 50 % reduction in growth rate compared to untreated controls [126]. NPs can cross and damage protective insect barriers, such as the cuticle or gut epithelium, primarily due to their ability to induce ROS and interact with critical cellular components [103,127]. These physical damages may result in part from the ROS induced by NPs, which suggests a synergistic interaction between structural (physical) injury and biochemical stress.

Smaller NPs can penetrate insect cells and midguts through mechanisms such as phagocytosis, transcytosis, endocytosis, and diffusion [128]. An in vitro study on *Spodoptera frugiperda* cells and isolated midguts indicated that peptide NPs were internalized via transcytosis, where they are enveloped in vesicles on one side of the cell, transported across, and released on the opposite side [131]. *Bombyx mori* treated with dietary Cu and ZnO NPs showed significant accumulation of Cu and Zn (with higher levels of Zn) in various tissues (Malpighian tubule, gut, fat body, and silk gland), with some NPs eliminated through feces. NP exposure also led to reduced larval body mass, survivorship, and cocoon production, with ZnO NPs having a stronger effect [129]. Histological examinations showed that nSiO_2_ exposure caused dose-dependent midgut tissue injury in silkworms, along with reduced larval body mass and cocoon production [130]. Other in vitro pulmonary toxicology studies on polyethylene terephthalate nanoplastic particles (less than 20 nm in size) have shown that NPs are internalized by cells, affect cell viability, and may interfere with the respiratory system at higher concentrations [132]. These entry mechanisms not only facilitate NP distribution but also prepare the internal cellular environment for subsequent disruptions, such as oxidative stress and apoptosis. Once inside the cell, NPs can alter membrane permeability and induce cell membrane damage, which is commonly associated with cytotoxicity. Ag-NPs have been shown to induce significant pathophysiological and ultrastructural changes in the ovarian tissues of darkling beetles (*T. molitor*), with exposure leading to disruptions in in tissue structure, altered cell function, oxidative stress, and apoptosis (programmed cell death) [133].

Studies on the effects of Ag NPs (50–60 nm) and ZnO NPs (10–30 nm) at nonlethal concentrations on the larval stages of *S. littoralis* indicated a reduction in larval and pupal weight. ZnO NPs also extended the larval developmental period, increased the counts of total hemocyte granular cell and plasmatocyte, and decreased levels of proteins, lipids, and carbohydrates. Ag NPs, in contrast, increased only plasmatocytes. ZnO NPs also enhanced enzyme activities, indicating interference with the digestive and immunological physiology and development of *S. littoralis* [103]. Biochemical modifications due to NPs include altered enzyme activity and modifications to protein structure and function. These changes may not occur in isolation but are closely linked to cellular damage caused by ROS and disruptions in other cellular activities, such as metabolism. ROS generated by NPs can disrupt cellular homeostasis and damage cellular components such as lipids, proteins, and DNA [134]. This interplay indicates a feedback mechanism where oxidative damage exacerbates metabolic dysregulation, further intensifying physiological stress on the organism. A study on *Blaps sulcata* (Coleoptera: Tenebrionidae) revealed increased copper accumulation 30 days after a single exposure to CuO NPs. The accumulation of CuO NPs in the testes of *Blaps sulcata* was associated with degenerative changes, apoptosis, DNA damage, dysregulation of the antioxidant system (increased superoxide dismutase (SOD) activity, decreased catalase (CAT) activity), and reduced acetylcholinesterase (AChE) activity [135]. The interaction between ROS and enzymatic dysregulation in these studies could suggest a feedback loop, where cellular damage contributes to metabolic disturbances, further destabilizing physiological homeostasis. In silkworms, exposure to nSiO_2_ nanoparticles caused structural and tissue injuries, induced antioxidant enzyme activities, and altered gene expression in pathways related to amino acid metabolism, lipid metabolism, and xenobiotic biodegradation. 16S rDNA sequencing revealed changes in gut microbial diversity, with decreased bacterial species richness and an increased prevalence of pathobionts (e.g., *Pseudomonas*, *Bacillus*, *Escherichia*, *Enterococcus*, *Ralstonia*) over probiotics (e.g., *Acetobacter*) [130]. Similarly, exposure to CuO and ZnO NPs disrupted nutrient metabolism (e.g., alpha-amylase), impaired antioxidant enzyme activities (e.g., SOD, GST, CAT), and affected gene expression (e.g., Attacin, lysozyme, SOD, Dronc), further contributing to oxidative stress and gut microbiome imbalances [129]. Exposure of *Acheta domesticus* to Ag NPs caused significant changes in digestive enzymes, including increased activities of α-amylase, α-glucosidase, and lipase but inhibited protease activity, while prolonged exposure to higher concentrations reduced food consumption, altered assimilation, and affected body weight in crickets [136]. A study conducted on wild-type and mutant *Drosophila* revealed that ZnO NPs did not affect melanin levels, phenol oxidase activity, or key metabolites in the melanin pathway [137,138]. A parallel study by the same author found that Ag2O NPs partially reversed depigmentation in *Drosophila* by minimally interacting with tyrosine, allowing melanin production, while strongly interacting with dopa, preventing its availability for melanization. This contrasted with the previous study on *Drosophila*, which showed no effect on melanin levels or pathway components [139]. Further researches should also investigate other metabolic pathways, such as the TOR (Target of Rapamycin) signaling pathway, a critical regulatory pathway that controls cell growth and metabolism.

Catalase, crucial for scavenging hydrogen peroxide (H_2_O_2_), showed a remarkable increase in activity in *B. mori* larvae during the last instar when exposed to 100 μg/mL ZnO-NPs, likely reflecting an adaptive response to elevated H_2_O_2_ levels. At 50 and 100 μg/mL concentrations, NPs significantly reduced different hemocyte counts (DHC) and total hemocyte count (THC), except for oenocytes, which showed a significant increase [140]. ZnO NPs induced oxidative stress in *C. pipiens*, as indicated by increased H_2_O_2_ levels. Detoxification responses to this included inhibited alkaline phosphatase (ALP) and glutathione peroxidase (GPX), activated SOD, and unchanged CAT activity [126]. Increased CAT activity with rising NP concentration was also observed in *S. oryzae* treatedwith biosynthesized TiO_2_ NPs [87].Chitosan NPs loaded with *Piper nigrum* essential oil (CS/PNO NPs) reduced the AChE activity of larvae of *S. oryzae* and *T. castaneum* [85]. Enzyme analysis after treating *S. litura* with Ag NPs derived from *O. basilicum* (ObAg NPs) showed a slight increase in carboxyl esterase and glutathione-S-transferase activity, although these changes were not statistically significant [141]. The effects of non-lethal concentrations of gold nanoparticles (Au-NPs) on ovarian tissue in *Trachyderma hispida* beetles were investigated, revealing significant reductions in antioxidant enzyme activities (GPOX and GSTP GPx) and suppressed transcription of miRNAs (miRNA-282 and miRNA-989), markers of genetic damage. Increased ovarian cell apoptosis was confirmed via TUNEL and annexin V-Fitc assays, and ultrastructure analysis highlighted pathological changes [135,142]. These findings emphasize the interconnected nature of physiological, biochemical, and molecular responses. Disturbances at the molecular and enzymatic levels—such as altered gene expression, suppressed antioxidant enzyme activities, and impaired nutrient metabolism—can trigger cascading systemic effects. Additionally, reductions in gut bacterial diversity, with a shift toward pathogenic microbes over probiotics, further exacerbate the physiological disruptions caused by NPs. Collectively, these findings underline the complex and interconnected nature of NP-induced physiological disruptions. Structural damage, oxidative stress, enzyme dysregulation, and microbial imbalance appear to act synergistically, leading to cascading effects that compromise insect development, reproduction, and survival. Future studies should focus on mapping these interdependencies to develop a more integrative understanding of NP mechanisms.

## 4 Efficacy and biosafety studies on NPs

Many studies report near or complete mortality, highlighting the significant potential of NPs as effective pest control agents. For instance, Bt-ZnO NPs achieved 100 % mortality of *C. maculatus* at 25 μg/mL (LC50: 10.71 μg/mL) within two weeks [107], while CuO NPs caused 55–94.4 % mortality in *S. granarius* at an LC90 of 300 mg/100 g of seed [18]. Similarly, nano silica and nano ZnO demonstrated 100 % mortality against *C. chinensis* at 1000 ppm within 5–7 days, and nano zeolite achieved 96.66 % mortality for *T. confusum* at 800 ppm after two weeks [103,108]. These findings underscore the high efficacy of NPs, although most results are derived from laboratory or controlled storage trials. The limited availability of field-scale studies emphasizes the need for further research to validate their safety, real-world applicability, and reliability.

Research on residue analysis of NPs for pesticidal use remains in its infancy, but preliminary studies suggest that green-synthesized NPs, such as Cu NPs and Ag NPs, exhibit lower toxicity profiles compared to conventional pesticides [46]. NPs could offer dual benefits by acting as pest control agents and enhancing agricultural outputs, such as seed germination and crop yield. However, understanding the concentrations and types of NPs that might pose risks remains critical. Further evaluations of residue analysis, safety for human and animal consumption, and impacts on beneficial insects are required to optimize formulations and ensure safe, large-scale adoption of NPs in storage pest control. The subsequent sections 4.1, 4.2, 4.3 examine findings from studies on the biosafety of NPs, focusing on their effects on seed germination, impact on non-target organisms, and dose-dependent and organ-specific effects of NPs.

### 4.1 Seed germination

The potential of NPs to positively influence seed germination has been highlighted in numerous studies. For instance, rice straw-based silica NPs not only achieved 100 % mortality of the pest *C. maculatus* at 200 ppm but also enhanced cowpea seed germination by 2 % [81]. Both Ag and Cu NPs, which exhibited 100 % mortality of pulse beetles at 1000 ppm, also improved seed germination and quality parameters [16]. Conversely, the study by Ref. [83] indicated that green NiO NPs at 40 ppm significantly reduced seed germination, suggesting that the impact on seed germination can vary significantly based on the type and concentration of NPs used. Additionally, SiO₂ NPs applied to the soil at up to 10 g/kg improved maize growth and yield, and combining 50 % mineral NPK (Nitrogen, Phosphorus, and Potassium) with 10 g/kg SiO_2_ NPs resulted in the most significant enhancement of agronomic traits, increasing protein content by 12.59 % and chlorophyll content by 45.13 SPAD [74]. Treatments with NSA also significantly reduced grain weight loss, frass production, and dirty grain effect [114].

A study by Ref. [112] indicated that non-biogenic ZnO NP treated maize seeds showed a higher (99 %) germination rate and greater (4.8 g) dry matter production, in addition to effectively controlling the pest *S. oryzae*. Seed priming with Si NPs significantly reduced cadmium (Cd) accumulation in wheat, improving growth, yield, photosynthesis, and antioxidant enzyme activity, while decreasing oxidative stress. Similarly, foliar spraying of Si NPs mitigated chromium (Cr) toxicity in rapeseed by regulating photosynthesis, oxidative stress, and antioxidant defense. The studies showed reduced metal uptake and enhanced plant resilience, suggesting Si NPs' potential for mitigating heavy metal toxicity and improving crop performance [143,144]. Green synthesized TiO_2_ nanoparticles from *Ricinus communis* L. leaf extract effectively mitigated Cr (VI) toxicity in plants, reducing oxidative stress and improving growth more than synthetic counterparts, indicating their potential as agricultural nano-based nutrition aids [145].

The phytotoxicity evaluation of commercially purchased Cu NPs, Potassium (KI) NPs, Ag NPs, Betadine (Bd) NPs, and Gentian (Gv) NPs concluded with a result that these NPs did not harm the morphology of corn plants after a two-week treatment, while exhibiting insecticidal activities against *S. frugiperda* larvae. Despite minor leaf spotting observed with KI NPs treatment, overall, the NPs did not adversely affect corn plant morphology in greenhouse experiments. Additionally, soil analysis showed no significant impact on pH or nutrient levels in the treated plots [71]. These findings indicate the dual benefits of NPs as the effective pest control and enhanced seed germination.

### 4.2 Impact on non-target organisms

In assessing the biosafety of NPs used as nanopesticides, it is crucial to consider their potential impact not only on the target pests but also on non-target organisms. A recent research highlights varying degrees of impact, such as the minimal toxicity of essential oil-loaded Polymeric NPs to non-target aquatic organism (*Artemia salina)* and osteoblast cells, while still demonstrating effective insecticidal properties against specific pests [84]. Similarly, biosynthesized TiO_2_ NPs demonstrated enhanced rice germination and insecticidal activity against the bean weevil, indicating nuanced effects that may depend on NPs’ characteristics like agglomeration [87]. These findings underscore the complex interactions and considerations needed for comprehensively evaluating the biosafety nanopesticides.

### 4.3 Dose-dependent and organ specific effects of NPs

The efficacy and safety of NPs as nanopesticides often depend on dosage. *B. thuringiensis*-derived ZnO NPs caused 100 % mortality of *C. maculatus* at a concentration of 25 μg/mL and significantly reduced mid-gut enzyme activity in a dose-dependent manner [107]. Similarly, nano silica and nano ZnO achieved 100 % pest mortality at 1000 ppm, with nano silica providing superior seed protection during storage compared to nano ZnO [108]. However, higher concentrations of Cu and ZnO NPs (100–2000 mg/L) have been shown to disrupt the nutritional balance of crops like mung beans, reducing the quality of phenolic compounds, macronutrients, and antioxidants, potentially posing risks to food safety and human health [146].

In another study, significant increases in *Acinetobacter* and *Enterococcus* in the gut microbiome were observed, showing strong correlations with predicted functional profiles and metabolite expressions, while histopathological evaluations provided insights into the internal effects of nanopesticides on organisms [147]. ZnO and SiO_2_ NPs were toxic to pests such as *S. oryzae* and *C. maculatus* [122]. Treated animals exhibited liver abnormalities, including congested blood sinusoids and binucleated hepatocytes, while lung tissues remained unaffected. This highlights the potential of nanopesticides to cause specific organ-related toxicity and emphasizes the need for detailed histopathological assessments. The limited scope of field-scale studies on nanopesticides, particularly concerning their effects on non-target plants, beneficial insects, and animals, as well as the safety of NP-treated seeds for human and animal consumption, necessitates extensive research to validate their practical applicability and ensure reliability in diverse real-world settings.

## 5 Advantages, challenges, and prospects of NPs in stored product pest control

A comprehensive analysis of recent studies illustrates both the advantages and challenges of NPs as nanopesticides. Table 1 presented an extensive review of studies illustrating the substantial efficacy of nanomaterials in pest control. Nanopesticides demonstrate exceptional targeting capabilities against stored product pests, often surpassing conventional chemical insecticides. Clove oil NPs maintained over 70 % mortality in *T. castaneum* for up to 16 weeks [105], while nano zeolite achieved complete mortality in *C. maculatus* within three days [103], a result that surpassed what was typically observed with conventional insecticides. Ag NPs not only controlled pests but also enhanced seed germination, suggesting potential value in agriculture [16]. In addition to their efficient targeting capabilities, NPs could also offer additional advantages by reducing environmental contamination and minimizing adverse effects on non-target organisms, including beneficial organisms [148]. Histopathological studies have suggested minimal effects on non-target organisms, implying that their careful integration into IPM strategies could significantly enhance the pest control efforts [122]. In a study by Ref. [46], green-synthesized Ag NPs were reported to degrade rapidly in the environment. This could lower their persistence in the environment and associated risks. Furthermore, as highlighted in the following section, nanopesticides could offer advantages over conventional pesticides by enhancing targeting capabilities, lowering effects on non-target organisms, integrating well with other pest control approaches, and offering eco-friendly pest control solutions.

Environmentally friendly green-synthesized NPs, such as Cu NPs derived from plant extracts, exhibited potent insecticidal activity with minimal environmental impact [46,148]. In addition, their ability to integrate with any of other pest control approaches maximizes their applicability in sustainable agriculture as combining NPs with chemical insecticides in some studies resulted in enhanced pest control efficacy and reduced chemical usage. For instance, Ag NPs combined with malathion achieved complete mortality of *T. castaneum* [37]. NPs like chitosan encapsulating essential oils ensured prolonged release and sustained insecticidal activity, thereby improving overall pest management efficiency and reducing the need for frequent applications [85]. A recent study showed that nano-chitosan-based insecticide nanoemulsions significantly enhanced the control of *S. littoralis* by improving stability, persistence, homogeneity, and efficacy [149]. Through reducing post-harvest losses, nanopesticides could contribute significantly to food security, particularly in regions prone to pest infestations during storage.

Despite their potential, several challenges remain for the widespread adoption of nanopesticides in stored product pest control. The lack of standardized synthesis methods and application protocols among researchers resulted in inconsistent findings regarding the overall efficacy of NPs in pest control, as illustrated in the table above. Diverse application methods further complicate efficacy comparisons, which would require targeted research to optimize formulations for specific pest groups. Variations in NPs’ characteristics, such as size, shape, and zeta potential, also pose challenges in establishing universal guidelines and dosage standards, as these factors significantly influence their outcomes. For example, Ag NPs at a concentration of 10 μg/mL enhanced the feed efficiency of *B. mori*, improved survivability, larval and pupal weights, and cocoon and shell weights; however, higher concentrations of certain NPs were seen to adversely affect non-target organisms [150]. Similarly, cobalt oxide NPs (Co_3_O_4_-NPs), despite being synthesized via an eco-friendly approach, exhibited dose-dependent phytotoxic effects and showed no insecticidal efficacy against major stored-product pests. These phytotoxic effects intensified with increasing concentrations, raising concerns about their potential impact on plant health in agricultural settings [111].

Moreover, regulatory hurdles pose significant barriers, as many countries lack clear guidelines for the approval and commercialization of NPs as insecticides. Future directions should prioritize the development of multifunctional NPs capable of delivering both pest control and plant nutrients through interdisciplinary research that integrates advances in nanotechnology with agricultural sciences. Additionally, the use of artificial intelligence to design tailored NP formulations with optimized efficacy and safety profiles represents a promising approach. Given that different reducing and stabilizing agents in NP synthesis result in varying insecticidal outcomes, targeted and standardized synthesis methods should receive greater attention. These challenges should be addressed through rigorous, collaborative research efforts focused on specific pest groups, as inconsistencies in synthesis and application may hinder the widespread adoption of nanopesticides. Biosafety assessments, particularly those evaluating the impacts of NPs on human health upon consumption, remain scarce and warrant immediate focus. Rigorous evaluations of bioaccumulation risks and ecological consequences, supported by stringent safety protocols and regulatory oversight, are essential to maximize the benefits of nanopesticides while minimizing potential risks to human health and the environment.

## 6 Conclusions

Nanoparticle-based insecticides could represent a highly efficient and innovative tool for controlling storage pests, with potential additional benefits such as enhanced seed germination, reduced toxicity, and rapid degradation in the environment. Extensive studies, as discussed earlier, on various NPs—such as alumina, carbon, silica, silver, copper, zinc oxide, nickel oxide, titanium dioxide, nano zeolite, chitosan, and polymeric NPs—have demonstrated significant insecticidal activity against both primary and secondary storage pests. Studies on various have demonstrated significant efficacy against pests such as *S. oryzae*, *T. castaneum*, and *C. maculatus*. These nanopesticides operate through mechanisms such as inducing oxidative stress, disrupting cellular functions, and causing structural damage, leading to insect mortality. However, the adoption of NPs in pest management necessitates addressing several challenges, including standardizing synthesis methods, conducting comprehensive biosafety studies, and understanding long-term ecological impacts. The potential for bioaccumulation and unforeseen consequences highlights the need for rigorous evaluation and regulatory oversight.

Integration of NPs with traditional insecticides have shown synergistic effects and improved pest control efficiency while potentially reducing chemical usage. However, such combinations should be approached with caution due to the harmful nature of some chemical insecticides. To fully realize the potential of nanopesticides in agriculture, future research should focus on optimizing NPs formulations, standardizing application methods, and ensuring their safety through extensive biosafety evaluations. By balancing scientific innovation with environmental stewardship, nanopesticides may potentially play a crucial role in advancing sustainable pest management practices, reducing post-harvest losses, and enhancing food security.

## CRediT authorship contribution statement

**Mohammed Lengichow Kadir:** Writing – review & editing, Writing – original draft, Visualization, Software, Formal analysis, Data curation, Conceptualization. **Asli Dageri:** Writing – review & editing, Writing – original draft, Formal analysis, Conceptualization. **Tuğba Nur Aslan:** Writing – review & editing, Writing – original draft, Formal analysis, Data curation, Conceptualization.

## Ethics statement

This article complies with the journal's ethics requirements. The manuscript does not report any research involving human participants, animals, or hazardous materials. All data used in this study are publicly available, and proper citations have been provided to acknowledge prior work.

## Data availability statement

All data generated or analyzed during this study are included in this article.

## Funding

This research did not receive any specific grant from funding agencies in the public, commercial, or not-for-profit sectors.

## Declaration of competing interest

The authors declare that they have no known competing financial interests or personal relationships that could have appeared to influence the work reported in this paper.
